# Spatial and Temporal Biogeography of Soil Microbial Communities in Arid and Semiarid Regions

**DOI:** 10.1371/journal.pone.0069705

**Published:** 2013-07-26

**Authors:** Zohar Pasternak, Ashraf Al-Ashhab, Joao Gatica, Ron Gafny, Shlomit Avraham, Dror Minz, Osnat Gillor, Edouard Jurkevitch

**Affiliations:** 1 Department of Microbiology and Plant Diseases, The Robert H. Smith Faculty of Agriculture, Food and Environment, The Hebrew University of Jerusalem, Rehovot, Israel; 2 Zuckerberg Institute for Water Research, The Jacob Blaustein Institutes for Desert Research, Ben-Gurion University of the Negev, Sede Boqer, Israel; 3 Forensic Biology Laboratory, Division of Identification and Forensic Science (DIFS), Israel Police National Headquarters, Jerusalem, Israel; 4 Institute of Soil, Water and Environmental Sciences, Agricultural Research Organization, The Volcani Center, Bet-Dagan, Israel; Université Paris Sud, France

## Abstract

Microbial communities in soils may change in accordance with distance, season, climate, soil texture and other environmental parameters. Microbial diversity patterns have been extensively surveyed in temperate regions, but few such studies attempted to address them with respect to spatial and temporal scales and their correlations to environmental factors, especially in arid ecosystems. In order to fill this gap on a regional scale, the molecular fingerprints and abundance of three taxonomic groups – Bacteria, α-Proteobacteria and Actinobacteria – were sampled from soils 0.5–100 km apart in arid, semi-arid, dry Mediterranean and shoreline Mediterranean regions in Israel. Additionally, on a local scale, the molecular fingerprints of three taxonomic groups – Bacteria, Archaea and Fungi – were sampled from soils 1 cm–500 m apart in the semi-arid region, in both summer and winter. Fingerprints of the Bacteria differentiated between all regions (P<0.02), while those of the α-Proteobacteria differentiated between some of the regions (0.01<P<0.09), and actinobacterial fingerprints were similar among all regions (P>0.05). Locally, fingerprints of archaea and fungi did not display distance-decay relationships (P>0.13), that is, the dissimilarity between communities did not increase with geographic distance. Neither was this phenomenon evident in bacterial samples in summer (P>0.24); in winter, however, differences between bacterial communities significantly increased as the geographic distances between them grew (P<0.01). Microbial community structures, as well as microbial abundance, were both significantly correlated to precipitation and soil characteristics: texture, organic matter and water content (R^2^>0.60, P<0.01). We conclude that on the whole, microbial biogeography in arid and semi-arid soils in Israel is determined more by specific environmental factors than geographic distances and spatial distribution patterns.

## Introduction

A large number of studies have demonstrated that microbial taxa display non-random environmental distribution. Particularly, bacterial communities may become increasingly different as the geographical distance between them increases [1]. Two mechanisms might contribute to this distance-decay relationship: the environmental conditions that are at play and bacteria dispersal limitation. The first mechanism may sort bacterial communities in accordance to their niche adaptations, which reflect specific environmental conditions. Hence, dissimilarity among microbial community compositions could match the disparity in environmental conditions, which, in turn, may be linked to geographical distances [2]. A wider view of this observation in the microbial world, namely that ‘everything is everywhere, but the environment selects’ [3], claims that spatial patterns of microbial diversity are driven by environmental heterogeneity rather than by dispersal limitation. Therefore, one might expect to find similar microbial communities in similar habitats and an assortment of microbial communities along an environmental gradient [4]. The second mechanism assumes that if individual bacterial dispersal (migration) is limited to adjacent sites, then spatial proximity will result in similar communities even if the sites are ecologically different. Thus, a distance-decay relationship would emerge even if the environmental conditions are comparable and the community niche requirements similar across regions [5].

In the past decade, a number of studies have centered on the biogeography of microbial species. These studies have indicated some degree of habitat endemicity [4], [6] at scales ranging from a few centimeters [7] to hundreds of kilometers [8], [9]. But although many microbial species might be globally distributed, using molecular techniques to identify microbial taxa, some surveys have shown that the distribution of microorganisms is also spatially limited in the same way as macroorganisms [10]. In fact, coupled with environmental data, the distance-decay relationship offers a mean to assess the relative importance of environmental heterogeneity and dispersal in controlling the spatial scaling of biodiversity. It is interesting to note that while microbial diversity patterns have been extensively surveyed, relatively few studies attempted to address microbial abundance patterns with respect to spatial and temporal scales and their correlations to environmental factors. Some studies have shown that environmental heterogeneity (and thus environmental selection) is the primary factor underlying microbial spatial differentiation [10]; still others pointed that the decline in the similarity of genetic structures of communities could be explained by geographic distance rather than by environmental heterogeneity, suggesting that dispersal limitation – especially at large spatial scales – is driving diversification [4].

Furthermore, most studies explored distance-decay relationships in temperate regions, while dry ecosystems were seldom investigated. Arid habitats are characterized by long periods of low water availability, drastic seasonal changes in plant cover and inputs of fresh organic matter, and large circadian temperature variation. These environmental characteristics likely have profound impacts on microbial communities, and this study addresses the gap in knowledge by exploring spatial and temporal patterns of the genetic structure of soil microbial communities in arid and semi-arid regions. We explored naturally occurring soil microbial communities at regional, local and diminutive scales, trying to correlate diversity and abundance to (i) ecosystem characteristics such as precipitation and soil features (type, organic matter content, etc.), and (ii) geographic distances.

## Materials and Methods

### 2.1. Soil sampling

**Figure 1:**
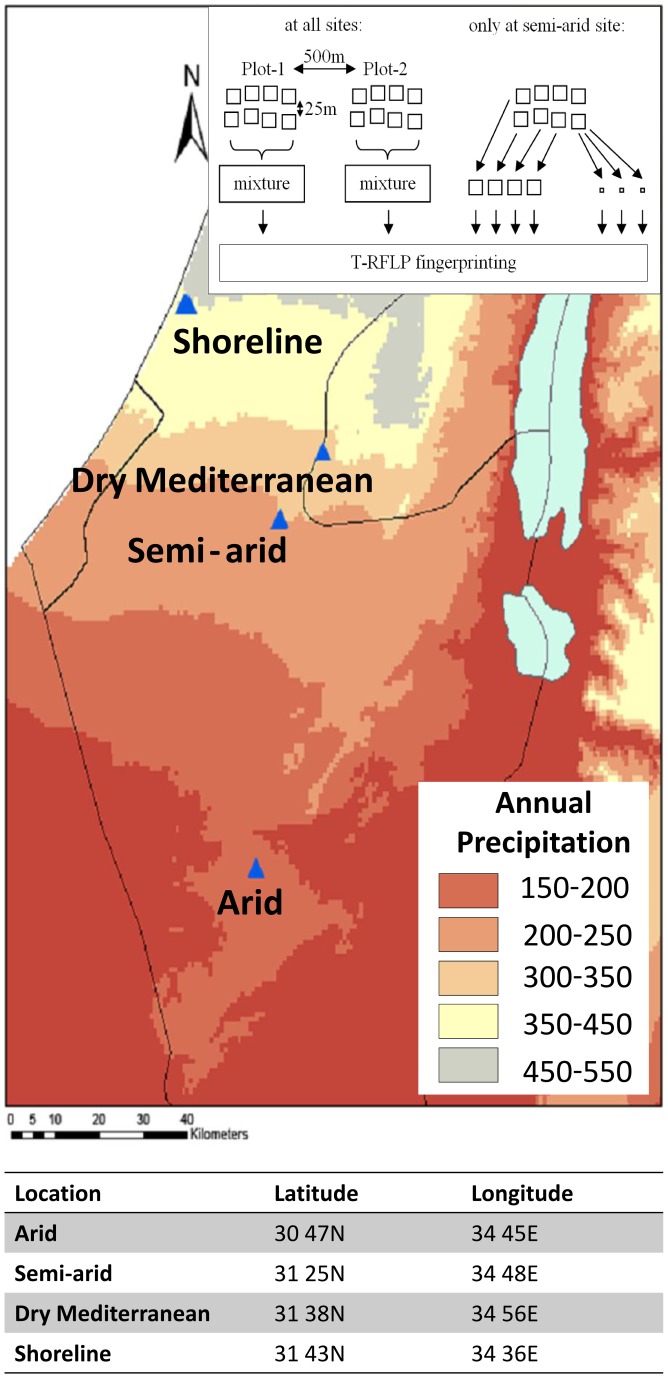
Long term ecological research (LTER) sites and the annual precipitation for the year 2009. Two replica plots were sampled in each of the arid, semi-arid and dry Mediterranean sites; in the shoreline site, five plots were sampled, representing three distinct soil types at close proximity. Additionally, at the semi-aris site, fingerprints of subsamples were also studied. Map and precipitation data from www.usgs.gov.

Six LTER (long-term ecological research; http://www.hamaarag.org.il/he/node/9) sites were sampled: three were similar in their soil silt content but different in their climatic characteristics (arid, semi-arid and dry Mediterranean), and three adjacent shoreline sites almost identical in their annual precipitation but different in their soil characteristics including shifting sand (1–10% vegetation cover), stabilized sand (30–60% vegetation cover) and alluvial red soil. The sites chosen for this study are illustrated in Fig. 1: dry Mediterranean (Adulam, 31 38N, 34 56E), 450 mm rain/year; shoreline (Nizzanim, 31 43N, 34 36E), 350 mm rain/year; semi-arid (Lehavim, 31 25N, 34 48E), 300 mm rain/year; and arid (Ovdat, 30 47N, 34 45E), 80 mm rain/year. No specific permits were required for the described field studies; the site locations are not privately-owned or protected, and the field studies did not involve endangered or protected species. Soil samples were taken from undisturbed, barren patches in plots that were fenced so that no livestock grazing or human activity occurred for at least five years prior to sampling. At each of the sites (except the alluvial shoreline site) two replica plots were chosen, ca. 500 m apart. Sampling took place during winter (March) and summer (August) of 2009. Therefore, 2 seasons ×5 sites ×2 plots  = 20 soil samples were taken, to which seasonal samples from a single plot of red alluvial seasonal soil were added, for a total of 22 samples. Each sample was an amalgam of eight 200 g sampling points that were randomly selected using a spatially stratified, random sampling grid. To compare microbial community structure at a local-scale, in addition to the samples separated by 500 m, the semi-arid site was further studied by examining four subsamples separated by about 25 m on the local random sampling grid. In addition, three small (4 g) soil samples a few cm from each other were retrieved from one of these subsites. All the samples in this study (from all sites) were collected aseptically from the top 5 cm of soil, from the nearest shrub inter-space patch, using ethanol-cleaned tools, after carefully brushing aside any loose litter and crust. The samples were placed in individual sterile plastic bags (Whirl-pak, USA), stored at 4°C in a cooler and transported to the laboratory where they were sieved and homogenized within 24 h and stored at −80°C awaiting molecular analysis.

### 2.2. Soil Physiochemical analysis

The following soil chemical and physical properties were measured using standard soil analytic methods [11]: soil water content, saturation percentage (by gravimetric method, in saturated soil extract – SSE), pH (in SSE), electrical conductivity (salinity; in SSE), sodium (in SSE), calcium and magnesium (in SSE by ICP (inductively coupled plasma) spectroscopy and flame photometer), sodium adsorption ratio (in SSE), percentage organic matter (by dichromate method), N as nitrate, N as ammonium (including adsorbed N), phosphorus in extract, potassium in extract, and soil texture – percentage of sand, silt and clay (by hydrometer and sieve method).

### 2.3. DNA extraction and amplification

Total nucleic acids (TNA) were extracted from each soil sample as previously described [12]. Some of the extracted DNA samples had heavy coloring indicating high concentration of contaminants that might inhibit the subsequent PCR reaction, thus these samples were further purified using MicroSpin S-200 HR columns (GE Healthcare, USA). The extracted nucleic acids (50 μl) were incubated at 37°C for 30 min with 1 μl RNAse and then the mixture was cleaned using DNA extraction Kit (Bioneer, S. Korea) according to the manufacturer's instructions. Table S1 describes the primers used for amplifying the 16S rRNA gene (Bacteria and Archaea) and the Internal Transcribed Spacer (ITS; Fungi). We have explored two taxonomic groups within the Bacteria, i.e. the Actinobacteria and α-Proteobacteria, as these were found to dominate in the explored soils [13], [14]. To minimize extraction bias, a composite of three DNA extractions from each sample were used as template, and each template was amplified by PCR in triplicate. The PCR mixture contained 200 nM of each PCR primer, 2.5 mM MgCl_2_, 0.8 μl of DreamTaq DNA polymerase (Fermentas, Canada), 5 μl DreamTaq buffer, 5 μl of bovine serum albumin solution (New England Biolabs, USA) and 0.2 mM of dNTPs (TaKara, Japan). Five μl of the PCR product were visualized on 1% agarose gel electrophoresis to ensure successful amplification.

### 2.4. Terminal restriction fragment length polymorphism (TRFLP)

PCR amplicons were treated with Mung bean exonuclease (TaKara, Japan) according to the manufacturer's instructions in order to eliminate ssDNA fragments that might result in pseudo-terminal restriction fragments (TRFs) [15]. The resulting dsDNA fragments were purified immediately using a PCR purification kit (Bioneer). DNA concentration was estimated by spectrophotometric analysis (NanoDrop, USA) and DNA was visualized on an agarose gel. Three different restriction enzymes (REs) per taxonomic group were used in order to increase the reliability of the TRFs' emerging patterns [16]. The REs were chosen using MiCA [17] and our in-silico restriction module, based on www.restrictionmapper.org: Bacteria – *HhaI*, *TaqI*, *HpyCH_4_IV*; Archaea – *MseI*, *MspI*, *AciI*; Fungi – *MnlI*, *MseI*, *AciI*; Actinobacteria – *HhaI*, *HapII*, *AciI*; α-Proteobacteria – *HhaI*, *HapII*, *HaeIII* (all REs were purchased from Fermentas except *HpyCH_4_IV* which was purchased from NEB). Enzymatic digestion was performed according to the specification of the manufacturers and was followed by purification using SigmaPrep™ spin column (Sigma, USA). The TRFs were visualized with ABI Prism® 3100 genetic analyzer (Applied Biosystems, USA). The peaks (TRFs) in each profile were related to specific fragment lengths based on a size marker (Liz500; Applied Biosystems) and visualized using Peak Scanner V.1.0 (Applied Biosystems). TRFs with base pair size below 40 and above 600 were truncated. ‘Noise’ peaks were filtered according to [18] and the true TRFs were aligned to the nearest integer. After alignment, matrixes were created for the peak height that indicated the relative abundance of each peak using T-REX (http://trex.biohpc.org) [19]. The above analysis was repeated for each RE and matrix attaching numbers to all ‘true’ peaks of TRFs indicating each peak's abundance and position for all the sites.

### 2.5. Real-Time PCR

To measure the abundance of each taxonomic group in the soil samples we used real-time, quantitative PCR (qPCR). Primers (Table S1) amplifying short fragments of maximum 450 bps were used in order to quantify the 16S rRNA copy numbers of Bacteria, Actinobacteria, and α-Proteobacteria. A calibration curve of known copy numbers of 16S rRNA genes were performed using DNA extracted from *Escherichia coli*, an actinobacterial isolate (Mareckova, unpublished data) and *Agrobacterium tumefaciens* were used to quantify Bacteria, Actinobacteria and α-proteobacteria, respectively. For calibration, five different standard serial dilutions were amplified in parallel to the samples. The range of qPCR efficiency was between 0.98–1.10. Triplicates of 25 μl were used for each qPCR reaction containing: 12 μl of DyNAmo Flash SYBR Green mix (Finnzymes, Finland); 6 μl of 200 nM primers; 2 μl of 10–20 ng μl^−1^ templates, and 5 μl of molecular grade water. Samples were run in a real time PCR machine (Corbett, Australia) under the following conditions: 40 cycles of 95°C for 10 sec, 58°C for 15 sec, 72°C for 20 sec. The relative abundance in each sample was calculated based on the calibration curves of the reference bacteria [13], [14].

### 2.6. Statistical analysis

The following analyses were performed using PC-ORD 5.32 (MjM, USA). Relativization was performed by sample total, i.e. each TRF abundance was relativized according to the total abundance of TRFs in that specific sample: b = X/(√ΣX_1..n_), where b is the relativized TRF abundance value, X is the original value, and n is the number of TRFs in that specific sample. Relativized values were square-root transformed, a procedure which increased the resolution by >10% (for a detailed account of the effects of transformation, see [20]). Community structures were compared using Bray-Curtis distances as the effect-size between zero and one: zero if the communities at the different sites were identical and one if the communities shared no TRFs. The statistical differences between soil fingerprints were measured using MRPP (multi-response permutation procedure) [21] and ANOSIM (analysis of similarity) [22] on the Bray-Curtis distance matrices. Ordinations were created using NMDS (non-metric multidimensional scaling) [23] on the Bray-Curtis distance matrices, and cluster analyses were performed with flexible beta (β = −0.25) using the Bray-Curtis distances.

## Results

### 3.1. Differentiation between soil microbial communities at the regional scale (15–100 km)

**Figure 2:**
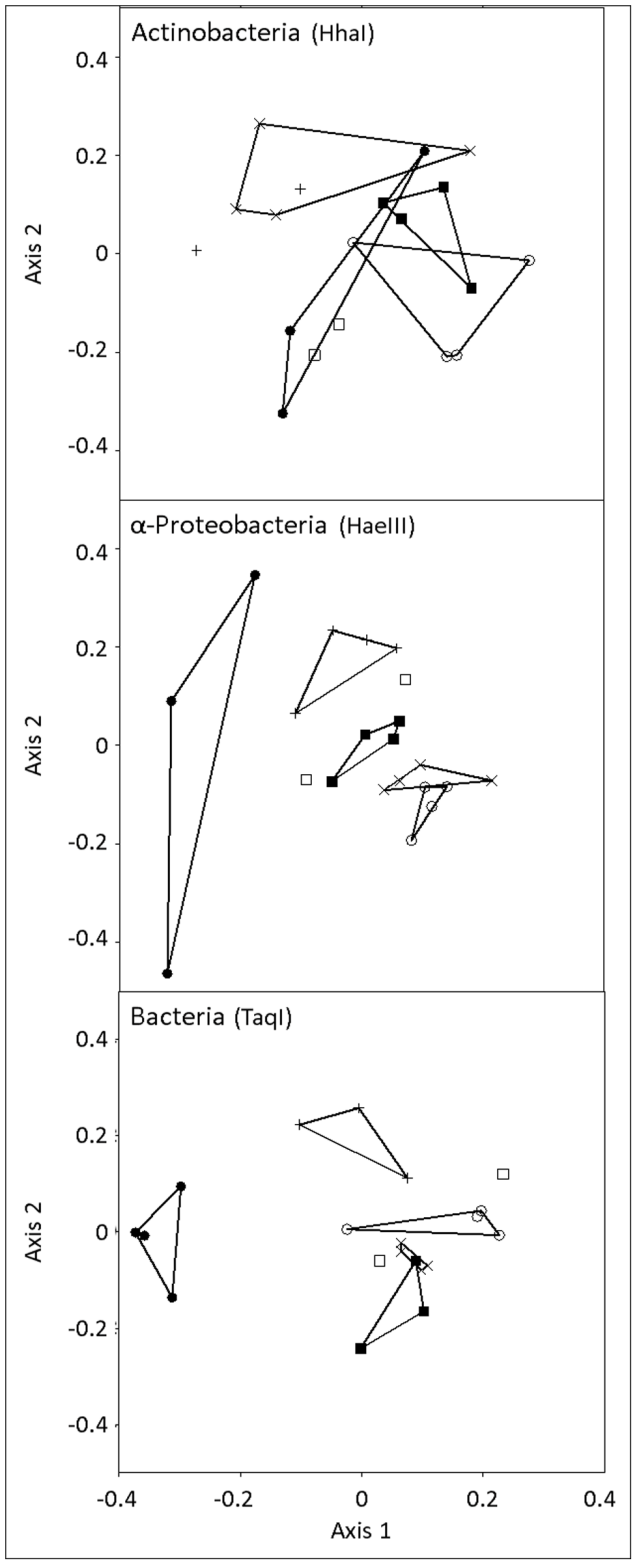
NMDS ordination of TRFLP fingerprints. Actinobacterial (top), α-proteobacterial (center) and bacterial (bottom) communities were sampled from dry Mediterranean (○), semi-arid (×), arid (▪), shoreline alluvial (□), shoreline stable sand (+) and shoreline shifting sand (•) sites. Restriction enzymes used are given in parentheses. Convex hulls connect samples from each site. NMDS axes do not possess biological meaning. Stress<12% for all ordinations, confirming a good correlation between the data and its ordination.

We examined three sites with a similar percentage of silt in the soil but different percentages of sand and clay, as well as different climatic characteristics, along a dry precipitation gradient (arid, semi-arid and dry Mediterranean). TRFLP fingerprints of three taxonomic groups – Bacteria, α-Proteobacteria and Actinobacteria – were taken from duplicate plots at each of the three sites in the summer and winter of 2009, exploring spatial and temporal differences (Figure 2). The Actinobacteria and α-Proteobacteria groups were found to dominate the bacterial community at the explored sites [13], [14] and were thus further investigated. For each taxonomic marker, the three restriction enzymes were compared (Table S2) and only the restriction enzymes with the highest spatial resolutions were further analyzed in this study. MRPP tests found that bacterial fingerprints significantly differed between all sites (P<0.02), whereas actinobacterial fingerprints differed between none of the sites (P>0.05). α-Proteobacteria were able to significantly differentiate between the arid and semi-arid (P = 0.02) and the arid and dry Mediterranean (P = 0.01) sites, but not between the semi-arid and dry Mediterranean (P = 0.09) sites. In addition, we tested the ability of the three taxonomic markers to differentiate between soil samples from different seasons within each site. Both α-proteobacterial and bacterial fingerprints showed no significant temporal differences (P>0.31), while actinobacterial fingerprints differed between summer and winter within each site (P<0.05).

### 3.2. Differentiation between microbial communities from adjacent sites with diverse soil types

The shoreline (‘Nizzanim’) site included samples from three different soil types collected at close proximity (1 km^2^): shifting sand, stabilized sand and alluvial red soil. TRFLP fingerprints of the three taxonomic groups – Bacteria, α-Proteobacteria and Actinobacteria – were taken from the three soil types in the summer and winter of 2009 in order to explore the effect of soil type on the microbial communities (Figure 2). MRPP tests found that both α-proteobacterial and bacterial fingerprints differed significantly between all three soil types (P<0.05), whereas actinobacterial fingerprints could not differentiate between shifting sand, stable sand and red soil (P>0.05).

### 3.3. Local (<1 km) and point-scale differentiation between microbial communities from similar soil types

**Figure 3:**
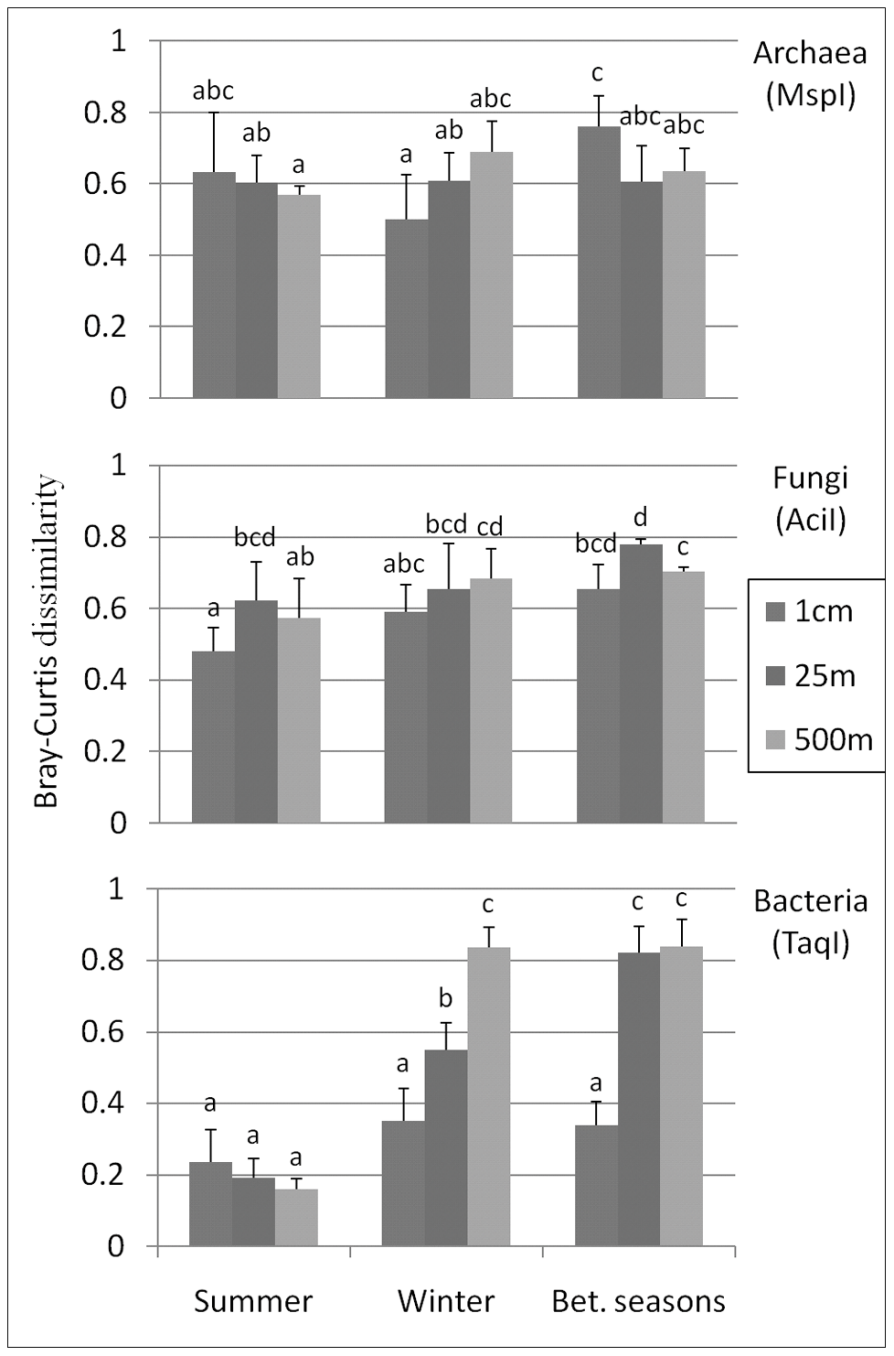
Differences in community structure between samples separated by different geographic distances and seasons. Bray-Curtis dissimilarity = 1 indicates that communities are very different, whereas Bray-Curtis dissimilarity = 0 indicates that communities are very similar. Each marker was studied using three restriction enzymes; only one is depicted here (and its name is given in parentheses), but for each marker, the overall pattern was very similar using the other two restriction enzymes (data not shown). Different letters indicate significant groupings. Bet., between.

Actinobacteria and, to a lesser extent, α-Proteobacteria displayed less discriminatory power than Bacteria (see above), because they were either (i) not sensitive enough to environmental conditions or (ii) too sensitive to local variations of environmental parameters; either way, using these taxonomic groups would restrict our ability to detect larger scale differences between samples as well as correlating these differences to abiotic factors, so we did not test these groups here; instead, we explored broader taxa, in the hope of acquiring better resolution power. The semi-arid soils were sampled at three spatial scales separated by 500 m, 25 m and 1 cm. TRFLP fingerprints of three taxonomic groups – Bacteria, Archaea and Fungi – were taken in duplicates from all semi-arid samplings in the summer and winter of 2009 in order to explore the effect of distance on the microbial communities. The distance-decay relationships of the Archaea and Fungal groups were not significant at the 1 cm – 500 m scales (MRPP tests, P>0.13); in other words, closer communities (geographically) were not more similar (Figure 3). Bacterial communities, however, behaved differently: in summer they showed high spatial homogeneity (Bray-Curtis distance<0.30) at all distances, while in winter they displayed a significant distance-decay relationship with differences between bacterial communities significantly increasing with geographic distance (P<0.01) (Figure 3).

### 3.4. Determinants of microbial community differentiation

**Figure 4:**
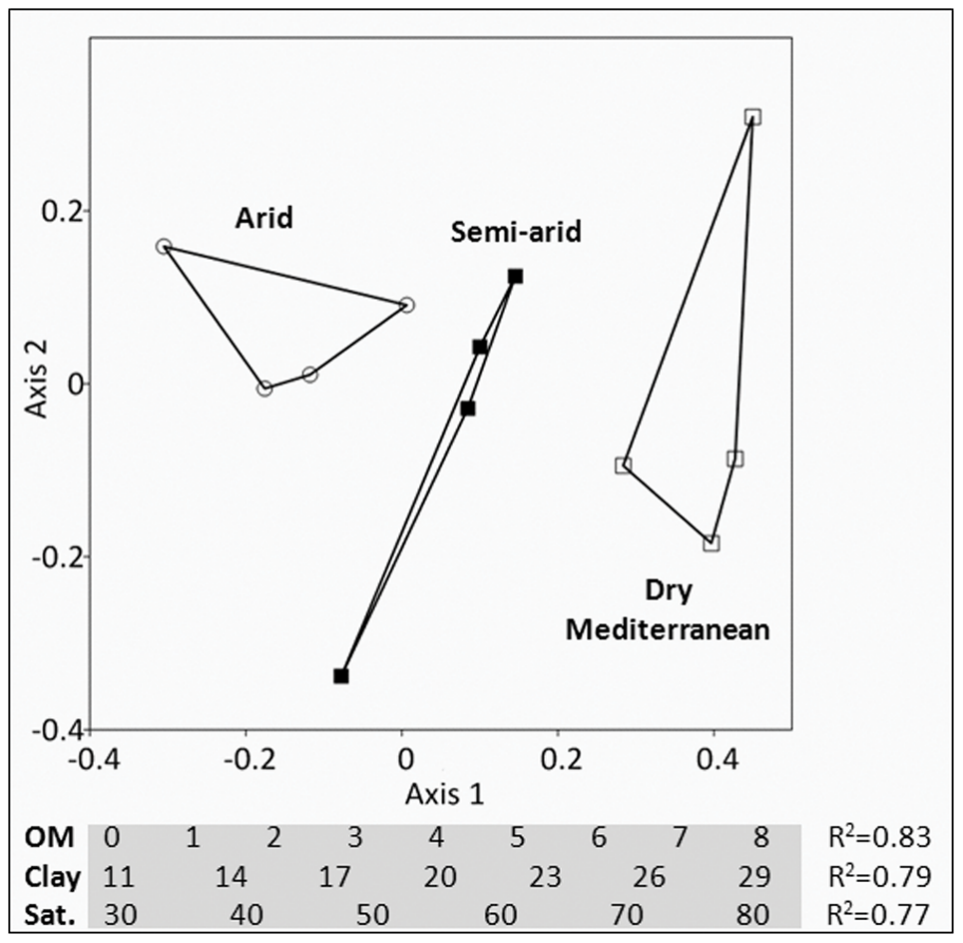
NMDS ordination of bacterial communities from arid, semi-arid and dry Mediterranean soils (stress = 13%). Closer points imply more similar communities. Bacterial communities differ significantly between the three locations (MRPP test, P<0.05). Axes of the ordination have no biological meaning; however, percentages of organic matter (OM), clay and water saturation (sat.) in the soils correlated well with axis 1 (see R^2^ values in graph) and can each reliably replace it.

Sixteen physical and chemical parameters were measured for each soil sample (Table 1) in the search of an explanation for the patterns of community structure of the Bacteria, α-Proteobacteria and Actinobacteria in the soil. In addition, the abundance of Bacteria, α-Proteobacteria and Actinobacteria was measured in each soil sample. The arid, semi-arid and dry Mediterranean soil bacterial community structure correlated with the organic matter content, clay/sand/silt composition, and water saturation in the soil (R^2^>0.77, P<0.01; Fig. 4). In the shoreline sand, pH emerged as an additional parameter that determines the bacterial community structure (R^2^ = 0.33, p<0.01). The α-Proteobacteria communities correlated to similar abiotic parameters, i.e. water, organic matter, sand and clay contents, which accounted each for more than two-thirds of the community variation (R^2^>0.67, P<0.01). Finally, the structures of Actinobacteria communities correlated to water, clay and phosphorous contents (R^2^>0.42, P<0.01).

**Table 1:** Main characteristics of the soils at the six sampling sites at winter and summer.

			WC	OM	K	P	Nh4	NO3	SAR	Mg	Ca	Na	EC	pH	Sat.	clay	silt	sand
**Shifting sand**	**winter**	**mn**	1.12	0.05	3.4	2.95	2.4	1.35	0.44	3.95	37.2	0.45	0.29	8.45	25	0.5	0.5	99
		**SD**	–	0	0.71	1.34	0	0.49	0.11	0.49	5.87	0.07	0.01	0.07	1.41	–	–	–
	**summer**	**mn**	0.05	0.07	3.6	2	2.71	2.4	0.84	6.4	53.2	1.05	0.45	8.4	24	0.5	0.5	99
		**SD**	–	0.01	0.85	0.99	1.45	0.57	0.3	0	12.8	0.49	0.15	0	0.71	–	–	–
**stable sand**	**winter**	**mn**	0.21	0.15	9.2	6.05	2.65	1.45	0.22	9.7	104	0.35	0.61	7.95	28.5	2	1	97
		**SD**	–	0.04	0	2.47	0.64	0.07	0.06	4.53	90.3	0.07	0.43	0.21	0.71	–	–	–
	**summer**	**mn**	0.1	0.07	10.7	4.35	3.91	2	0.28	9.9	76.7	0.45	0.45	7.7	27	2	1	97
		**SD**	–	0.03	1.34	0.07	1	0.28	0.06	0.85	1.77	0.07	0.01	0	1.41	–	–	–
**alluvial soil**	**winter**	**mn**	14.96	4.47	19	24.9	14.7	3.7	0.65	26.6	171	1.5	1.01	7.7	65	35	39	26
		**SD**	–	–	–	–	–	–	–	–	–	–	–	–	–	–	–	–
	**summer**	**mn**	3.22	2.18	11.7	7.4	10.2	3.1	0.37	30.3	242	1	1.1	7.2	54	35	39	26
		**SD**	–	–	–	–	–	–	–	–	–	–	–	–	–	–	–	–
**Arid**	**winter**	**mn**	4.05	0.16	20.3	4.7	2.55	1.45	1.46	49.6	312	5.7	1.8	8.1	30	12	55.5	32.5
		**SD**	–	0.15	23.1	3.82	0.21	0.64	1.56	64	383	7.5	2.11	0.42	5.66	1.41	6.36	4.95
	**summer**	**mn**	2.14	0.48	14	12.9	6.34	3.35	2.4	13.6	76.9	3.9	0.89	8.1	30	12	55.5	32.5
		**SD**	–	0.45	1.41	1.48	0.47	0.35	1.92	3.75	14.7	3.39	0.52	0	0	1.41	6.36	4.95
**semi-arid**	**winter**	**mn**	7.72	2.19	34.3	4.85	7.4	7.8	2.22	85.5	787	10.6	4.23	7.5	52.5	17.5	59	23.5
		**SD**	–	0.04	2.26	0.64	0.85	2.69	0.75	2.4	51.8	3.32	0.04	0	0.71	0.71	4.24	3.54
	**summer**	**mn**	1.99	1.84	9.15	3.7	11.2	7.9	0.87	19.3	165	1.9	0.99	7.7	50	17.5	59	23.5
		**SD**	–	0.17	1.77	0.85	0.43	2.4	0.22	0.14	9.33	0.42	0.03	0.14	2.83	0.71	4.24	3.54
**dry med**	**winter**	**mn**	23.42	6.71	8.15	6.1	11.7	2.85	0.16	18.5	246	0.45	1.12	7.55	73.5	27	60	13
		**SD**	–	0.02	0.78	0	1.48	0.35	0	5.09	49.2	0.07	0.21	0.07	2.12	2.83	1.41	1.41
	**summer**	**mn**	3.85	7.04	8.2	9.55	26.1	8.3	0.3	23.1	299	0.85	1.28	7.2	81.5	27	60	13
		**SD**	–	1.02	2.26	0.78	0.01	0.28	0.05	4.45	38.7	0.21	0.18	0.14	2.12	2.83	1.41	1.41

Values are mean (mn) ± standard deviation (SD, where available) of two duplicate plots at each station. WC, water content (%); EC, electrical conductivity (dS/m); NO_3_, nitrate (mg/kg); P, phosphorus (mg/kg); K, potassium (mg/kg); Na, sodium (mg/kg); Ca, calcium (mg/kg); Mg, magnesium (mg/kg); SAR, sodium adsorption ratio; OM, organic matter (%); NH_4_, ammonium (mg/kg); Sat., saturation; clay, silt, sand, % of soil particles.

### 3.5. Microbial abundance

Actinobacterial abundance (i.e. number of cells) did not follow a spatial pattern, as this group's abundance was similar at all three sites – arid, semi-arid and dry Mediterranean. The abundance of total Bacteria and α-Proteobacteria correlated with precipitation: smaller communities were present at the arid site than at the semi-arid site, and they reached higher concentrations at the dry Mediterranean site (Table S3). In addition to precipitation, the abundance of the Bacteria and α-Proteobacteria was also significantly and positively correlated to soil water saturation, organic matter content and soil texture (R^2^>0.6, P<0.04). Differences in bacterial abundance were not significantly correlated to the geographic distance between the samples (R^2^ = 0.23, P = 0.44) or to the microbial communities of soils collected from the same site (R^2^ = 0.15, P = 0.19).

### 3.6. Environmental resolution of microbial fingerprints

**Figure 5:**
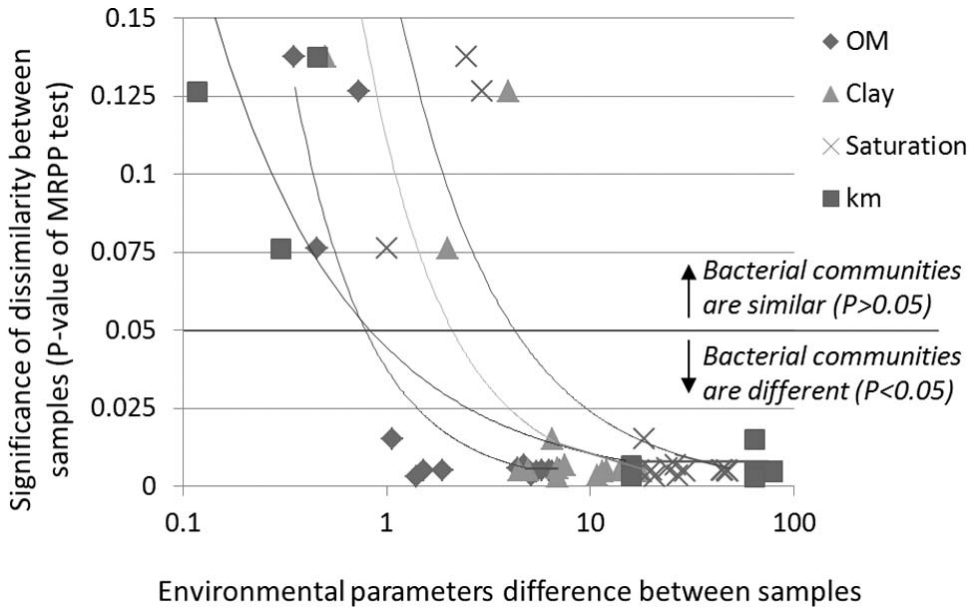
Higher differences in environmental parameters cause higher differences in community structure. Each point in the graph represents a comparison between two samples: the X axis depicts the difference between the two samples with respect to the following environmental parameters in soils: OM – organic matter; Clay – clay percentage; Saturation – water saturation; km – geographic distance between the samples in kilometers; the Y axis is the significance of the dissimilarity (MRPP tests' P-value) between bacterial communities from the two samples. Trend lines are R^2^>0.8 for all environmental parameters. Values under the ‘0.05 line’ signify two bacterial communities that are significantly different (P<0.05).

The higher the **s**imilarity in the environmental characteristics of soils the smaller was the likelihood to detect differences in their microbial fingerprints (Fig. 5). Based on this correlation, the resolution of microbial fingerprints in soil can be defined as the minimal environmental difference that allows for two communities to be differentiated at a P = 0.05 level. Here, a difference of at least 0.71 percentage points in organic matter content was sufficient to yield significant and detectable differences in bacterial community (Fig. 5). Similarly, when applied to soil water saturation and clay content, values of 8.8 and 3.7 percentage points were obtained, respectively. The minimal resolution in terms of geographic distance was 830 m; however, this value is only relevant where the compared soil samples come from areas with similar soil parameters and precipitation.

## Discussion

The major goal of this study was to understand the causes of microbial community structure at spatial and temporal scales by examining relative contributions of distance and environment over seasons. This study thus links local and regional spatial scales, and temporal scales, to the structure of microbial communities examined using Bacteria, Actinobacteria, α-Proteobacteria, Archaea and Fungi. It has been previously shown that under spatial gradients ranging from few centimeters to few meters [7], hundreds of meters [24] and to continental scales [2], [8] bacterial communities in samples from proximate soils were significantly more similar than those from samples collected farther apart. It had also been documented that environmental heterogeneity affects bacterial community composition more than geographical distance [4]. Temporal variations, climatic change and precipitation gradients were also shown to alter bacterial diversity and community composition [25], [26], but not the total bacterial biomass [27]; [28]. Plant and microbial communities in the soil may be connected, i.e. a different botanical community will contribute toward creating a different microbial community, thus obscuring interpretation of results. Therefore, our soil samples at all the regions were taken from fenced, barren patches – land plots devoid of any plant cover, that for the past five years have not experienced animal grazing or human activity.

The community structures of soil samples taken from different regions along the precipitation gradient differed completely when using a bacterial marker, less when examining the α-Proteobacteria, and not at all when analyzing the Actinobacteria. Additionally, when the total cell abundance was counted, bacterial and α-proteobacterial populations in soil increased with the rise in precipitation but the Actinobacteria did not. Thus, actinobacterial communities remained stable in both structure and quantity throughout large distances along the precipitation gradient in dry regions, as well as differences in the sand and clay contents of the soil. This may be due to the ability of sporulating as well as non-sporulating actinobacterial species to survive high drought conditions [29], [30] or even grow in dry soil [31]. In contrast, α-Proteobacteria are more abundant under milder conditions in the same soils during the same season [14]. It is noteworthy that in some of our analyses, only restriction enzymes (RE) that showed the highest spatial resolution were used, and it is possible that eliminating the data of lower-resolution RE might have biased the results. However, for every T-RFLP experiment there are hundreds of potential RE one can use, and a choice has to be made; invariably, the researcher chooses the “best” (or “highest resolution”) RE out of many, and this study is no exception.

Our data from soils in arid and dry regions, on the whole, did not show significant distance-decay relationships. This statement is true for almost all geographic distances, seasons, taxonomic groups and TRFLP restriction enzymes; for example on a regional scale, the semi-arid site is geographically closer to the dry Mediterranean site (35 km) than it is to the arid site (65 km), but its bacterial community is more similar to that in the arid site than it is to the dry Mediterranean (and the same pattern is seen with α-proteobacteria). The only distance-decay relationship found was the bacterial fingerprints at the local spatial scale (25 m–500 m) of the semi-arid region in winter. These results suggest that overall, the level of similarity between microbial communities in dry soils could not be solely explained by geographical distance [32]. Alternatively, it could be attributed to the differences found between climatic regions and soil types/textures. Furthermore, for several of the taxonomic groups, proximate (centimeters apart) semi-arid soil samples were found to host distinct bacterial communities, while soils collected many meters away from each other were similar. In contrast, soils collected a few kilometers apart in distinct sites (corresponding to shifting and stabilized sand at the shoreline region) yielded distinct bacterial communities suggesting specialization to each unique habitat. These results follow Sensabaugh [33] who suggested that some changes in the composition of microbial populations are expected to occur over short distances of millimeters or centimeters. Moreover, it was shown that grains of soil may hold highly variable and distinct bacterial communities [34].

We have shown that on a local scale, spatial distribution patterns hold for Bacteria but not for Archaea or Fungi i.e. that distance decay relationships could not be detected for these two groups over local spatial scales. Likewise, a previous study conducted in fragmented forests in Panama showed weak spatial scale dispersal in mycorrhizal Fungi [35]. We propose that differences in soil microbial fingerprints could best be explained by changes in environmental factors, in our case precipitation [18], organic matter and soil texture. Marked differences in soil organic content and soil texture resulted in statistically significant differences between microbial communities (Fig. 4), whereas temporal patterns had limited effect on bacterial diversity and abundance. A study exploring the diversity of salt marsh bacteria over centimeters to continental spatial scale found that environmental factors overruled geographic distance, having crucial effects on community similarity within the salt marshes but no detectable effect at larger scales [2]. For instance, within marshes moisture explained community similarity, while water temperature and nitrate concentrations were more important at the regional and continental scales, respectively. Our study suggests that when soils are characterized by distinct environmental factors, each will likely host a unique community of microorganisms, regardless of the geographic distance between them. In arid soils, these factors are moisture, organic matter, and silt/clay content. An emerging picture combining ours and others' results is that the main drivers shaping the soil microbiota may change according to the climatic regions and the scale of the analysis [2], [32], [36], requiring sampling at local scales to uncover them.

## Supporting Information

Table S1 **PCR primers and procedures used in this study.**
(DOCX)

Table S2 **Comparison of three restriction enzymes (RE) for TRFLP analysis using each of the five microbial markers.** For each RE, the power to differentiate between sites was analyzed as the A-statistic of the MRPP test. For each marker, the best RE (i.e. highest A) was set as 100%, and the differentiation power of the other two RE was calculated as percentage of the best RE (called A%). Asterisk denotes significant differentiation between sites (MRPP test, P<0.05).
(DOCX)

Table S3 **Abundance of α-proteobacterial, actinobacterial and bacterial 16S rRNA.** Values are mean and standard deviation (SD) of two qPCR measurements in each season.
(DOCX)

## References

[1] Nekola JC, White PS (1999) The distance decay of similarity in biogeography and ecology. J Biogeog 26: 867–878.

[2] Martiny JBH, Eisen JA, Penn K, Allison SD, Horner-Devine MC (2011) Drivers of bacterial beta-diversity depend on spatial scale. Proc Nat Acad Sci USA 108: 7850–7854. 21518859 10.1073/pnas.1016308108 PMC3093525

[3] Baas-Becking LGM (1934) Geobiologie of inleiding tot de milieukunde. WP Van Stockum & Zoon, The Hague, the Netherlands.

[4] Green J, Bohannan BJM (2006) Spatial scaling of microbial biodiversity. Trends Ecol Evol 21: 501–507. 16815589 10.1016/j.tree.2006.06.012

[5] Bell T (2010) Experimental tests of the bacterial distance-decay relationship. Isme J 4: 1357–1365. 20535220 10.1038/ismej.2010.77

[6] Martiny JBH, Bohannan BJM, Brown JH, Colwell RK, Fuhrman JA, et al (2006) Microbial biogeography: putting microorganisms on the map. Nature Rev Microbiol 4: 102–112. 16415926 10.1038/nrmicro1341

[7] Franklin RB, Mills AL (2003) Multi-scale variation in spatial heterogeneity for microbial community structure in an eastern Virginia agricultural field. Fems Microbiol Ecol 44: 335–346. 12830827 10.1016/S0168-6496(03)00074-6

[8] Cho JC, Tiedje JM (2000) Biogeography and degree of endemicity of fluorescent *Pseudomonas* strains in soil. Appl Environ Microbiol 66: 5448–5456. 11097926 10.1128/aem.66.12.5448-5456.2000 PMC92480

[9] Griffiths RI, Thomson BC, James P, Bell T, Bailey M, et al (2011) The bacterial biogeography of British soils. Environ Microbiol 13: 1642–1654. 21507180 10.1111/j.1462-2920.2011.02480.x

[10] Nemergut DR, Costello EK, Hamady M, Lozupone C, Jiang L, et al (2011) Global patterns in the biogeography of bacterial taxa. Environ Microbiol 13: 135–144. 21199253 10.1111/j.1462-2920.2010.02315.x PMC5834236

[11] Page AL, Miller RH, Keeney DR (1982) Methods of soil analysis, Part 2: chemical and microbiological properties. American Society of Agronomy, Madison, WI

[12] Angel R, Matthies D, Conrad R (2011) Activation of methanogenesis in arid biological soil crusts despite the presence of oxygen. Plos One 6. 10.1371/journal.pone.0020453 PMC3105065 21655270

[13] Bachar A, Al-Ashhab A, Soares M, Sklarz M, Angel R, et al (2010) Soil microbial abundance and diversity along a low precipitation gradient. Microb Ecol 60: 453–461. 20683588 10.1007/s00248-010-9727-1

[14] Bachar A, Soares MIM, Gillor O (2012) The effect of resource islands on abundance and diversity of bacteria in arid soils. Microb Ecol 63(3): 694–700. 22038034 10.1007/s00248-011-9957-x

[15] Egert M, Friedrich MW (2003) Formation of pseudo-terminal restriction fragments, a PCR-related bias affecting terminal restriction fragment length polymorphism analysis of microbial community structure. Appl Environ Microbiol 69: 2555–2562. 12732521 10.1128/AEM.69.5.2555-2562.2003 PMC154551

[16] Moyer CL, Dobbs FC, Karl DM (1994) Estimation of diversity and community structure through restriction fragment length polymorphism distribution analysis of bacterial 16S rRNA genes from a microbial mat at an active, hydrothermal vent system, Loihi Seamount, Hawaii. Appl Environ Microbiol 60: 871–879. 7512808 10.1128/aem.60.3.871-879.1994 PMC201404

[17] Shyu C, Soule T, Bent SJ, Foster JA, Forney LJ (2007) MiCA: a web-based tool for the analysis of microbial communities based on terminal-restriction fragment length polymorphisms of 16S and 18S rRNA genes. Microb Ecol 53: 562–570. 17406775 10.1007/s00248-006-9106-0

[18] Angel R, Soares MIM, Ungar ED, Gillor O (2010) Biogeography of soil archaea and bacteria along a steep precipitation gradient. Isme J 4: 553–563. 20033070 10.1038/ismej.2009.136

[19] Culman SW, Bukowski R, Gauch HG, Cadillo-Quiroz H, Buckley DH (2009) T-REX: software for the processing and analysis of T-RFLP data. Bmc Bioinformatics 10. 10.1186/1471-2105-10-171 PMC2702334 19500385

[20] Pasternak Z, Al-Ashhab A, Gatica J, Gafni R, Avraham S, et al (2012) Optimization of molecular methods and statistical procedures for forensic fingerprinting of microbial soil communities. Int Res J Microbiol 3(11): 363–372.

[21] Mielke P (1984) Meteorological applications of permutation techniques based on distance functions. Elsevier Science Publishers, 813–830.

[22] Clarke KR, Green RH (1988) Statistical design and analysis for a biological effects study. Mar Ecol Prog Ser 46: 213–226.

[23] Mather PM (1976) Computational methods of multivariate analysis in physical geography. John Wiley and Sons, Inc, London.

[24] Horner-Devine MC, Lage M, Hughes JB, Bohannan BJM (2004) A taxa-area relationship for bacteria. Nature 432: 750–753. 15592412 10.1038/nature03073

[25] Frey SD, Drijber R, Smith H, Melillo J (2008) Microbial biomass, functional capacity, and community structure after 12 years of soil warming. Soil Biol Biochem 40: 2904–2907.

[26] Moore-Kucera J, Dick RP (2008) PLFA profiling of microbial community structure and seasonal shifts in soils of a Douglas-fir chronosequence. Microb Ecol 55: 500–511. 17786504 10.1007/s00248-007-9295-1

[27] Bell CW, Acosta-Martinez V, McIntyre NE, Cox S, Tissue DT, et al (2009) Linking microbial community structure and function to seasonal differences in soil moisture and temperature in a chihuahuan desert grassland. Microb Ecol 58: 827–842. 19466479 10.1007/s00248-009-9529-5

[28] Clark J, Campbell J, Grizzle H, Acosta-Martinez V, Zak J (2009) Soil microbial community response to drought and precipitation variability in the chihuahuan desert. Microb Ecol 57: 248–260. 19067031 10.1007/s00248-008-9475-7

[29] Goodfellow M, Williams ST (1983) Ecology of actinomycetes. Ann Rev Microbiol 37: 189–216. 6357051 10.1146/annurev.mi.37.100183.001201

[30] LeBlanc JC, Gonçalves ER, Mohn WW (2008) Global response to desiccation stress in the soil actinomycete *Rhodococcus jostii* RHA1. Appl Environ Microbiol 74: 2627–2636. 18326668 10.1128/AEM.02711-07 PMC2394902

[31] Williams ST, Shameemullah M, Watson ET, Mayfield CI (1972) Studies on the ecology of actinomycetes in soil – VI: The influence of moisture tension on growth and survival. Soil Biol Biochem 4(2): 215–225.

[32] Fierer N, Jackson RB (2006) The diversity and biogeography of soil bacterial communities. Proc Nat Acad Sci USA 103: 626–631. 16407148 10.1073/pnas.0507535103 PMC1334650

[33] Sensabaugh G (2009) Microbial community profiling for the characterization of soil evidence: forensic considerations, In: al Re (Ed), Criminal and environmental soil forensics. Springer Science and Business Media, USA, 49–60.

[34] Vetsigian K, Jajoo R, Kishony R (2011) Structure and evolution of streptomyces interaction networks in soil and in silico. PLoS Biol 9. 10.1371/journal.pbio.1001184 PMC3201933 22039352

[35] Mangan SA, Eom AH, Adler GH, Yavitt JB, Herre EA (2004) Diversity of arbuscular mycorrhizal fungi across a fragmented forest in Panama: insular spore communities differ from mainland communities. Oecologia 141: 687–700. 15322901 10.1007/s00442-004-1684-2

[36] Lauber CL, Hamady M, Knight R Fierer N (2009) Pyrosequencing-based assessment of soil pH as a predictor of soil bacterial community structure at the continental scale. Appl Environ Microbiol 75: 5111–5120. 19502440 10.1128/AEM.00335-09 PMC2725504

