# Supplementary material for: Spatial and Temporal Biogeography of Soil Microbial Communities in Arid and Semiarid Regions
Source: PLoS One. 2013 Jul 26;8(7):e69705. doi: 10.1371/journal.pone.0069705 (PMC3724898; doi:10.1371/journal.pone.0069705)
Supplement: Table S1 — PCR primers and procedures used in this study. (DOCX) [file pone.0069705.s001.docx]

| Target domain | Temperature cycling | Primer sequences (5'->3') | References |
| --- | --- | --- | --- |
| Bacteria | 30 cycles of 45 S @ 94°C, 1 min @ 45°C, 3 min @ 72°C. | 341: CCTACGGGAGGCAGCAG | ([Ishii and Fukui, 2001](#_ENREF_20); [Liu et al., 1997](#_ENREF_24)) |
|  |  | 907: CCGTAATCMTTTGAGTT |  |
| Actino-bacteria | 30 cycles of 45 S @ 94°C, 1 min @ 51°C, 3 min @ 72°C. | S-C-Act235-a-S-20: CGCGGCCTATCAGCTTGTTG | ([Kyselkova et al., 2008](#_ENREF_22); [Stach et al., 2003](#_ENREF_37)) |
|  |  | 16Sact1114r: GAGTTGACCCCGGCRGT |  |
| α-Proteobacteria | 30 cycles of 45 S @ 94°C, 1 min @ 45°C, 3 min @ 72°C. | ADF-681F: AGTGTAGAGGTGAAATT | ([Blackwood et al., 2005](#_ENREF_8)) |
|  |  | ADF-685R: TCTACGRATTTCACCYCTAC |  |
| Archaea | 30 cycles of 60 S @ 94°C, 60 S @56°C, 60 S @72°C. | 344: ACGGGGCGCAGCAGGCGCGA | ([Vissers et al., 2009](#_ENREF_40)) |
|  |  | 915: GTGCTCCCCCGCCAATTCCT |  |
| Eukarya  (Fungi) | 35 cycles of 60 S@95°C, 2 min@56°C, 2 min@72°C. | ITS1: CTTGGTCATTTAGAGGGAAGTA | ([Vancov and Keen, 2009](#_ENREF_38)) |
|  |  | ITS86: TTCAAAGATTCGATGATTCAG |  |
