# Supplementary material for: Spatial and Temporal Biogeography of Soil Microbial Communities in Arid and Semiarid Regions
Source: PLoS One. 2013 Jul 26;8(7):e69705. doi: 10.1371/journal.pone.0069705 (PMC3724898; doi:10.1371/journal.pone.0069705)
Supplement: Table S2 — Comparison of three restriction enzymes (RE) for TRFLP analysis using each of the five microbial markers. For each RE, the power to differentiate between sites was analyzed as the A-statistic of the MRPP test. For each marker, the best RE (i.e. highest A) was set as 100%, and the differentiation power of the other two RE was calculated as percentage of the best RE (called A%). Asterisk denotes significant differentiation between sites (MRPP test, P<0.05). (DOCX) [file pone.0069705.s002.docx]

| **Marker** | **RE** | **A%** |
| --- | --- | --- |
| **Bacteria** | TaqI | 100.0^*^ |
|  | HhaI | 92.5^*^ |
|  | HaeIII | 78.3^*^ |
| **Actinobacteria** | HhaI | 100.0^*^ |
|  | HapII | 25.7 |
|  | AciI | 6.6 |
| **α-proteobacteria** | HaeIII | 100.0^*^ |
|  | HhaI | 46.7^*^ |
|  | HapII | 15.9^*^ |
| **Archaea** | MspI | 100.0^*^ |
|  | MseI | 97.7^*^ |
|  | AciI | 32.3 |
| **Fungi** | AciI | 100.0^*^ |
|  | MnlI | 25.1^*^ |
|  | MseI | 16.9^*^ |
