# Supplementary material for: Spatial and Temporal Biogeography of Soil Microbial Communities in Arid and Semiarid Regions
Source: PLoS One. 2013 Jul 26;8(7):e69705. doi: 10.1371/journal.pone.0069705 (PMC3724898; doi:10.1371/journal.pone.0069705)
Supplement: Table S3 — Abundance of α-proteobacterial, actinobacterial and bacterial 16S rRNA. Values are mean and standard deviation (SD) of two qPCR measurements in each season. (DOCX) [file pone.0069705.s003.docx]

|  | **Winter** | | **Summer** | |
| --- | --- | --- | --- | --- |
| **Alphaproteobacteria** | **Mean** | **SD** | **Mean** | **SD** |
| Dry Mediterranean | 6.61E+04 | 1.43E+04 | 6.58E+05 | 2.40E+04 |
| Semi-arid | 5.20E+04 | 3.24E+03 | 9.77E+04 | 2.18E+03 |
| Arid | 3.09E+04 | 5.76E+03 | 1.26E+04 | 6.15E+03 |
| Alluvial | 9.35E+04 | 9.19E+02 | 7.20E+04 | 1.34E+03 |
| Stabilized sand | 9.64E+02 | 4.60E+02 | 1.17E+03 | 6.48E+01 |
| Stratified sand | 2.59E+02 | 5.85E+01 | 3.99E+02 | 1.19E+02 |
| **Actinobacteria** | **Mean** | **SD** | **Mean** | **SD** |
| Dry Mediterranean | 6.29E+04 | 1.05E+04 | 4.04E+04 | 1.26E+04 |
| Semi-arid | 6.45E+04 | 1.15E+04 | 3.65E+04 | 8.04E+03 |
| Arid | 5.99E+04 | 2.99E+02 | 3.76E+04 | 7.28E+03 |
| Alluvial | 8.97E+03 | 1.03E+03 | 9.42E+03 | 5.87E+02 |
| Stabilized sand | 5.70E+02 | 1.70E+02 | 9.11E+02 | 5.40E+02 |
| Stratified sand | 4.78E+02 | 2.80E+01 | 2.09E+02 | 2.54E+01 |
| **Bacteria** | **Mean** | **SD** | **Mean** | **SD** |
| Dry Mediterranean | 4.87E+09 | 1.70E+01 | 3.05E+09 | 2.04E+08 |
| Semi-arid | 2.49E+09 | 3.39E+00 | 1.94E+09 | 1.53E+07 |
| Arid | 1.41E+09 | 5.94E+01 | 1.30E+09 | 5.77E+07 |
| Alluvial | 3.00E+09 | 8.49E+00 | 2.76E+09 | 6.52E+08 |
| Stabilized sand | 4.94E+08 | 2.34E+01 | 5.49E+08 | 8.32E+07 |
| Stratified sand | 1.62E+07 | 2.97E+01 | 1.84E+07 | 3.21E+06 |
